# Supraventricular Tachycardia Beyond the Heart: A Case of Undiagnosed Graves' Thyrotoxicosis Presenting as an Episode of Supraventricular Tachycardia

**DOI:** 10.7759/cureus.85594

**Published:** 2025-06-09

**Authors:** Muhammad Haroon Riasat, Aparna Ravikumar, Samraiz Nafees, Shahzad Akbar, Khush Bakht

**Affiliations:** 1 Diabetes and Endocrinology, York and Scarborough Teaching Hospitals NHS Foundation Trust, Scarborough, GBR; 2 Internal Medicine, York and Scarborough Teaching Hospitals NHS Foundation Trust, Scarborough, GBR; 3 Endocrinology and Diabetes, Hull University Teaching Hospitals NHS Trust, Hull, GBR; 4 Cardiology, Hull University Teaching Hospitals NHS Trust, Hull, GBR

**Keywords:** endocrine system disorder, graves's disease, management of thyrotoxicosis, supraventricular tachycardia (svt), thyroid screening

## Abstract

Thyrotoxicosis is a hypermetabolic condition defined by elevated levels of triiodothyronine (T3) and/or thyroxine (T4) in the serum. While irregular heart rhythms like atrial fibrillation are commonly linked to this condition, other types such as supraventricular tachycardia (SVT) characterized by sudden onset, narrow QRS complexes, and regular RR intervals in the absence of structural heart disease are much less frequently reported and often overlooked.

We present a compelling case of Graves’ thyrotoxicosis in a middle-aged male patient, whose only presenting symptom was SVT, in the absence of typical systemic signs of hyperthyroidism or structural cardiac abnormalities. This case challenges the conventional diagnostic trajectory of thyrotoxicosis, emphasizing the importance of including thyroid function testing in the diagnostic workup of unexplained arrhythmias.

Early recognition of such atypical presentations is crucial, as appropriate treatment of the underlying thyrotoxicosis can lead to resolution of the arrhythmia and improved clinical outcomes.

## Introduction

Graves’ disease is one of the most common causes of endogenous hyperthyroidism, accounting for the majority of cases in iodine-sufficient regions [1]. It is characterized by the presence of autoantibodies directed against the thyroid-stimulating hormone (TSH) receptor, which act as agonists and induce excessive secretion of thyroid hormones. This autoimmune stimulation effectively disengages the thyroid gland from hypothalamic-pituitary regulation, resulting in sustained thyrotoxicosis [2] and typically presents with classical symptoms such as diffuse goiter, ophthalmopathy, weight loss, heat intolerance, and tremors. However, clinical manifestations can be highly variable, ranging from subtle subclinical hyperthyroidism with minimal symptoms to severe systemic involvement [1,2].

Thyroid hormones exert broad systemic effects, with particularly profound influence on cardiovascular physiology. Acting through both genomic and non-genomic pathways, triiodothyronine (T3) and thyroxine (T4) enhance myocardial contractility, increase heart rate, and reduce systemic vascular resistance, culminating in a hyperdynamic circulatory state [1,3]. Since the earliest clinical descriptions of thyrotoxicosis, cardiovascular symptoms have consistently served as prominent and often alarming features, prompting medical evaluation [3].

Common cardiovascular manifestations include palpitations, exertional dyspnea, angina-like chest pain, peripheral edema, systolic hypertension, a hyperdynamic point of maximal cardiac impulse, and cutaneous vasodilation [3]. These signs collectively reflect the pervasive effects of thyroid hormone excess on both the electrical and mechanical functions of the heart. Among rhythm disturbances associated with hyperthyroidism, sinus tachycardia is most frequently encountered, often followed by atrial fibrillation, particularly in patients over the age of 60 [2,3]. Their occurrence as an isolated and initial presentation, particularly in the absence of structural cardiac abnormalities, is infrequent and diagnostically challenging, with limited documentation in the literature [3].

Herein, we describe a case of Graves’ thyrotoxicosis without the classic signs of thyrotoxicosis like tremors, Goitre or Graves' ophthalmopathy, in which supraventricular tachycardia (SVT) was the sole presenting feature. This case underscores the importance of considering thyroid dysfunction in patients with unexplained arrhythmias, even in the absence of overt systemic signs of hyperthyroidism.

## Case presentation

**Figure 1:**
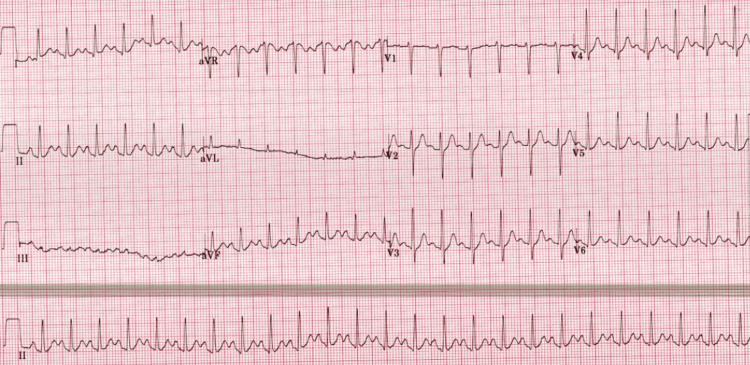
ECG at the Time of Presentation Showing Supraventricular Tachycardia with Narrow QRS Complexes, Regular R-R Intervals, and a Heart Rate of 154 Beats/min

A middle-aged man with no significant past medical history presented to the emergency department with an acute onset of palpitations and a sensation of chest tightness. On initial evaluation, the patient appeared visibly anxious, restless, and fidgety. Vital signs revealed a blood pressure of 165/102 mmHg and a heart rate of 154 beats per minute, as shown in Figure 1. Electrocardiography demonstrated a regular narrow complex tachycardia consistent with SVT.

The patient reported no prior cardiovascular diagnoses, denied substance use, and described himself as physically active, frequently participating in competitive cycling events. He did, however, disclose two recent episodes of transient loss of consciousness over the preceding few weeks, both preceded by marked palpitations.

Laboratory investigations revealed normal serum electrolytes, including calcium, magnesium, potassium, and phosphate. Inflammatory markers were unremarkable (C-reactive protein <1 mg/L), and high-sensitivity troponin levels were mildly elevated but equivocal (19 ng/L, normal <4.9 ng/L), as shown in Table 1. There was no clinical or biochemical evidence of infection or electrolyte imbalance to explain the acute onset of SVT.

**Table 1:** Patient's Initial Laboratory Investigations at Presentation

Laboratory Tests	Results	Reference Ranges
Hemoglobin	145 g/L	130-180 g/L
White Cell Count	11.1 *10^9/L	4.0-11.0 *10^9/L
CRP	<1 mg/L	0-5 mg/L
Serum Magnesium	0.88 mmol/L	0.70-1.00 mmol/L
Serum Calcium	2.22 mmol/L	2.20-2.60 mmol/L
Serum Phosphate	0.85 mmol/L	0.80-1.5 mmol/L
Urea	3.9 mmol/L	2.5-7.8 mmol/L
Creatinine	83 umol/L	59-104 umol/L
Serum Sodium	134 mmol/L	133-146 mmol/L
Serum Potassium	4.1 mmol/L	3.5-5.3 mmol/L
1^st^ Troponin	19 ng/L	<4.9 ng/L
2^nd^ Troponin	19 ng/L	<4.9 ng/L

Initial management included administration of intravenous fluid boluses and beta-adrenergic blockade with bisoprolol, which resulted in effective rate control, symptomatic improvement, and restoration of sinus rhythm. Given the absence of a clear structural or metabolic trigger, a comprehensive laboratory evaluation was undertaken. Thyroid function tests revealed a profoundly suppressed thyroid-stimulating hormone (TSH) level of <0.01 mIU/L, with markedly elevated free thyroxine (T4: 54 pmol/L, normal: 11-22 pmol/L) and triiodothyronine (T3: 39 pmol/L, normal: 3.1-6.8 pmol/L), consistent with a thyrotoxic state. Subsequent immunologic testing confirmed the presence of autoimmune thyroid disease, with elevated TSH receptor antibodies (TRAb: 15.9 IU/L, normal: 0.00-1.7 IU/L), as shown in Table 2. In collaboration with the cardiology service, a transthoracic echocardiogram was performed to evaluate for structural cardiac pathology. The echocardiogram revealed a preserved left ventricular ejection fraction (>55%), normal biventricular systolic function, and no morphological abnormalities of the cardiac valves. Initial investigations had revealed a mildly elevated troponin level; these troponin levels were not indicative of myocardial infarction, given the absence of dynamic changes on serial testing and lack of corresponding ischemic symptoms, ECG changes, or wall motion abnormalities on echocardiograph.

**Table 2:** Thyroid Function Tests

Laboratory Tests	Results	Reference Ranges
Thyroid-Stimulating Hormone	<0.01 mIU/L	0.27-4.20 mIU/L
T4 (Tetraiodothyronine)	54 pmol/L	11-22 pmol/L
T3 (Triiodothyronine)	39 pmol/L	3.1-6.8 pmol/L
TSH receptor antibodies (TRAb)	15.9 IU/L	0.00-1.7 IU/L

Based on these findings, a diagnosis of Graves’ thyrotoxicosis was established, with SVT as the initial and sole clinical manifestation. The patient was promptly initiated on antithyroid therapy with carbimazole 40 mg in consultation with the endocrinology team, alongside continued beta-blocker therapy for rate control. Following initiation of treatment, the patient experienced a notable improvement in symptoms and heart rate stability. No further episodes of SVT were documented on the telemetry after initiation of regular beta blockade whilst in-patient. Arrangements were made for follow-up with Cardiology and Endocrinology Teams, as well as outpatient Holter monitoring and coordination with primary care services to ensure comprehensive ongoing evaluation. No further syncopal or arrhythmic episodes were reported during the first month of follow-up.

This case highlights an uncommon presentation of Graves’ disease, where SVT occurs in the absence of structural cardiac disease or classical systemic symptoms of thyrotoxicosis. While atrial fibrillation is the more commonly observed arrhythmia in hyperthyroid states, narrow complex tachycardia as an initial presentation remains rare, with limited documentation in the literature. This underscores the critical importance of including thyroid function assessment in the diagnostic workup of unexplained supraventricular arrhythmias.

## Discussion

Graves’ disease is an autoimmune thyroid disorder defined by the presence of TRAb, which aberrantly activate the TSH receptor, leading to unchecked synthesis and release of thyroid hormones [4]. The resulting thyrotoxicosis exerts profound effects on multiple organ systems, with the cardiovascular system being among the most prominently affected.

While sinus tachycardia and atrial fibrillation are the most frequently encountered arrhythmic manifestations of hyperthyroidism, occurring in approximately 10-25% of cases, SVT remains an uncommon and underrecognized presentation [5,6]. The literature contains limited documentation of SVT as an isolated and initial manifestation of thyrotoxicosis, rendering such presentations diagnostically challenging and potentially life-threatening if unrecognized [6,7].

Thyroid hormones modulate cardiovascular physiology through complex genomic and non-genomic pathways [8]. Triiodothyronine (T3), the biologically active form of thyroid hormone, exerts its cellular effects primarily through nuclear receptor binding, regulating the transcription of genes responsible for myocardial structure and function [9]. Although thyroxine (T4) is the predominant hormone secreted by the thyroid gland, it is T3, either directly secreted or converted peripherally, that binds to cardiac nuclear receptors. Notably, cardiomyocytes lack significant deiodinase activity, rendering them dependent on circulating T3 for their hormonal signaling [5].

Once inside the cardiac myocyte, T3 influences transcription of numerous cardiac genes, particularly those regulating intracellular calcium handling and contractile protein synthesis. This includes upregulation of sarcoplasmic reticulum Ca²⁺-ATPase and its modulator phospholamban, key proteins responsible for calcium uptake during diastole. In addition, T3 enhances expression of fast isoforms of myosin heavy chains, sodium-potassium ATPase, and the sodium-calcium exchanger, factors that collectively augment myocardial contractility and excitability [5,9].

Cardiac pacemaker activity, governed by specialized myocytes of the sinoatrial node, is also modulated by thyroid hormone through both direct genomic effects and interactions with the autonomic nervous system. T3 influences transcription of hyperpolarization, activated cyclic nucleotide-gated (HCN) channels, namely HCN2 and HCN4, which regulate diastolic depolarization and pacemaker automaticity [8-10]. Simultaneously, thyroid hormones sensitize β-adrenergic receptors, enhance adenylate cyclase activity, and increase intracellular cyclic AMP levels, further accelerating heart rate and increasing susceptibility to arrhythmogenesis [8-10].

In addition to their direct myocardial actions, thyroid hormones induce systemic vasodilation, reduce systemic vascular resistance, and increase resting heart rate and cardiac output, all of which can precipitate or exacerbate cardiac dysrhythmias [9]. Furthermore, they modulate the autonomic nervous system by increasing sympathetic tone and decreasing vagal activity, thus contributing to the development of tachyarrhythmias [9].

The β-adrenergic signaling cascade is particularly responsive to thyroid hormone. Components such as the β1-adrenergic receptor and downstream effectors including adenylate cyclase are transcriptionally upregulated, explaining the efficacy of β-blockers in ameliorating both the hemodynamic and electrophysiological manifestations of hyperthyroidism [11].

In the present case, SVT was the primary and isolated manifestation of Graves’ thyrotoxicosis in the absence of structural heart disease or classical systemic symptoms of hyperthyroidism. The normalization of cardiac rhythm following initiation of antithyroid therapy and β-blockade underscores the pivotal role of thyroid hormone excess in arrhythmia pathogenesis. Given the rarity of such presentations, this case reinforces the need for clinicians to maintain a high index of suspicion for occult thyroid dysfunction in patients presenting with unexplained supraventricular tachyarrhythmias.

## Conclusions

This case illustrates a clinically significant presentation of Graves’ disease, where SVT emerged as the sole initial manifestation in a previously healthy, athletic individual with no structural cardiac abnormalities. While atrial fibrillation is the more frequently recognized arrhythmia in thyrotoxic states, SVT remains underreported and may be overlooked. It is essential for clinicians to consider thyroid dysfunction in the differential diagnosis of unexplained tachyarrhythmias, particularly when conventional etiologies such as structural heart disease, infection, and electrolyte imbalance have been excluded. Early recognition and treatment of the underlying thyrotoxic state, in conjunction with appropriate cardiac management, can result in full recovery of cardiac rhythm and function.
